# HIV viral suppression after enhanced adherence counselling in children on dolutegravir-based regimens in Malawi

**DOI:** 10.4102/sajhivmed.v26i1.1692

**Published:** 2025-05-09

**Authors:** Lucky Makonokaya, Maggie Khumbanyiwa, Louiser U. Kalitera, Rachel Chamanga, Shalom Dunga, Lilian Jiah, Elton Masina, Nilesh B. Bhatt, Thulani Maphosa

**Affiliations:** 1 Elizabeth Glaser Pediatric AIDS Foundation, Lilongwe, Malawi; 2 Elizabeth Glaser Pediatric AIDS Foundation, Washington, DC, United States of America

**Keywords:** enhanced adherence counselling, children living with HIV, viral suppression, HIV, dolutegravir, Malawi

## Abstract

**Background:**

Viral suppression (VS) rates in children on antiretroviral therapy (ART) are lower than in adults. We described the effect of enhanced adherence counselling (EAC) on VS among children with high HIV viral load accessing dolutegravir (DTG)-based ART in a programme setting in Malawi.

**Objectives:**

This study evaluated the proportion of children with high viral load on DTG-based ART who re-suppressed following EAC and factors associated with VS post-EAC.

**Method:**

We included all patients aged < 15 years with a high viral load result (> 1000 copies/mL) after taking DTG-based ART for at least 6 months between January 2022 and March 2023, covering 104 healthcare facilities in Malawi. Descriptive statistics summarised the distribution of demographic and clinical characteristics. Multivariable logistic regression determined the factors associated with VS following EAC.

**Results:**

Overall, 1475 participants were enrolled; 884 (59.9%) were aged 10–14 years. A total of 1448 (98.2%) were enrolled in EAC, of whom 787 (54.3%), 308 (21.3%), and 353 (24.4%) completed three, two, and one session(s), respectively. Follow-up HIV viral load results were available for 1091 (74%) participants enrolled in EAC, and 782 (71.7%) achieved VS. Patients from urban areas were less likely to achieve VS post-EAC than those from rural areas (adjusted odds ratio [aOR]: 0.58, 95% confidence interval [CI]: 0.42–0.80).

**Conclusion:**

Nearly three-quarters of children with high viral load on DTG-based regimens achieved VS following EAC. Further research on the other contributors of virologic failure among children in Malawi is required.

**What this study adds:** This study highlights the importance of EAC among children with virologic failure on DTG-based ART in Malawi, showing that nearly three-quarters achieved suppression following EAC. The study found lower rates of VS post-AC in urban populations, indicating the need for additional support.

## Introduction

Recently, the scale-up of dolutegravir (DTG)-based antiretroviral therapy (ART) regimens in children living with HIV (CLHIV) has led to improved viral suppression (VS).^1,2,3^ However, these significant gains still fall short of the ambitious 95% target set by the UNAIDS 95-95-95 initiative, which aims to end HIV as a public health threat by 2030.^4,5^ Globally in 2023, 66% of CLHIV knew their status, 57% initiated ART, and 48% were virally suppressed.^6^ Timely ART initiation, along with achieving VS, is crucial in people living with HIV (PLHIV), especially for children, as ART enhances the immune system’s recovery, slows HIV disease progression, and reduces morbidity and mortality.^7,8,9,10,11,12^ Poor adherence is a major cause of viral non-suppression in paediatric ART clients.^1,2,13,14^ Various individual and environmental factors, such as lack of HIV status disclosure within the household, inadequate support from the parents or guardians, and school-related pressures, have been associated with poor ART adherence in paediatric HIV patients.^15,16,17^

Enhanced adherence counselling (EAC) is an intervention that helps CLHIV and their caregivers identify their barriers to good ART adherence and develop ways to overcome the barriers sustainably. EAC plays a vital role in the care and treatment cascade, with approximately 70% of patients who complete the programme achieving VS, and is recommended by the World Health Organisation (WHO).^18,19^ While EAC has improved adherence and VS rates in adults living with HIV, tailoring this intervention to CLHIV is challenging, as counselling is mainly administered to caregivers rather than children themselves.

Malawi is a low-income country in southern Africa with an estimated population of 21.5 million as of 2024, 49% of which is under 15 years of age.^20,21^ CLHIV in Malawi lag behind adults in meeting 95-95-95 UNAIDS targets.^22^ In 2021, Malawi transitioned all children on first-line ART to DTG-based regimens when paediatric formulations became available; this transition was done regardless of VS status and without changing the nucleoside backbone of the regimen.^23^ Notably, while over 95% of adults on ART have achieved VS, only about 80% of children have achieved this milestone.^1^ The country’s Ministry of Health recommends EAC in patients on DTG-based ART with suspected treatment failure.^24^

There is limited research on the role of the EAC intervention on VS among CLHIV in a programme setting in Malawi, particularly in the era of optimised ART regimens. We sought to assess the effect of EAC on VS among children receiving DTG-based regimens in the country.

## Research methods and design

### Study design and setting

We conducted a retrospective cohort study using routinely collected data in the ART programmes’ electronic medical records system (EMRS) and viral load registers from 104 primary and secondary healthcare facilities which the Elizabeth Glaser Pediatric AIDS Foundation supports in the central and southern regions of Malawi. We included all children (age < 15 years) on DTG-based regimens in the selected health facilities who had unsuppressed viral loads between January 2022 and March 2023.

### Diagnosis and management of unsuppressed viral load

Unsuppressed viral load was defined as HIV RNA of 1000 copies/mL or higher. According to the Ministry of Health guidelines, routine viral load testing in children on adult formulations was done at 6 months, 12 months, and then every 12 months thereafter, while children on paediatric formulations were tested every 6 months following initiation or transition to DTG-based regimens.^25^ All ART patients with unsuppressed viral load results were entered into the high viral load registers. The patients were traced via phone call or physically for communication of their results and they were enrolled in EAC. Experienced healthcare workers, who were predominantly nurses, clinical officers, psychosocial counsellors, or treatment supporters trained in adherence counselling, conducted the EAC sessions using EAC standard operating protocols.^25^ The guidelines recommended at least one quality EAC session, though the frequency and duration of the sessions were determined by the counsellor, on an individual case basis. A repeat HIV viral load test was indicated 3 months after the first EAC session, if one or two sessions were required, or 1 month after the third session if three sessions were required. Adherence was assessed using pill count. For patients who remained virally unsuppressed following EAC sessions showing improved adherence, an HIV drug resistance (DR) testing application form was completed and sent via email to an expert drug resistance committee. Samples for HIV DR testing were collected and sent to the National Health Laboratory Service, Johannesburg, South Africa, upon approval from the committee.^26^ HIV-1 viral load and DR testing were conducted according to the manufacturer’s instructions as part of the routine testing procedures. Documentation of the number of EAC sessions, repeat viral load sample collection and results, and HIV DR testing was recorded in the high viral load registers. All viral load results were transcribed into the EMRS and the National Lab Information Management System (NLIMS).

### Data collection and statistical analysis

We collected the data in March 2024 using a digital data collection tool in the Microsoft Excel 2019 database (Microsoft, Redmond, WA, United States) and exported it to Stata^®^ version 17.0 (StataCorp LP, College Station, TX, United States) for analysis. Data were abstracted from facility-based high viral load registers and the EMRS, anonymised at the point of abstraction. We used a pre-set significance level of 0.05. Differences in categorical variables were compared using Pearson’s Chi-square test. Descriptive statistics were used to summarise patient demographic and clinical characteristics. Factors associated with post-EAC VS were assessed using a multivariable logistic regression, adjusting for gender, age group, duration on ART, facility location, initial ART regimen, and number of EAC sessions completed. All the covariates were included in the univariable and multivariable analysis.

### Patient and public involvement

Neither patients nor the public were involved in the design, conduct, reporting, or dissemination plans of the study.

### Ethical considerations

This evaluation was part of a protocol titled ‘Evaluation of Outcomes Achieved through Integrated HIV/AIDS and TB Prevention, Care, and Treatment Programs in Malawi’. Permission and ethical clearance to conduct the study was obtained from the Malawi National Health Sciences Research Committee (protocol number 18/09/2130) on 12 September 2018, and the Advarra Institutional Review Board (IRB) in the United States (protocol number Pro00040441) on 22 November 2019. This activity was reviewed by the United States Centers for Disease Control and Prevention (CDC), Lilongwe, Malawi and the CDC Atlanta, Georgia, and was conducted consistently with the applicable federal law and CDC policy. We obtained a waiver for the individual informed consent requirement because routinely collected data from medical records were being retrospectively evaluated and there were no interactions with the study participants.

## Results

Overall, 1475 participants were enrolled, half (725, 50.8%) being girls. The median age was 11 years (interquartile range [IQR]: 7–13), and 884 (59.9%) were aged 10–14 years. About two-thirds (68.3%) of the participants were from rural areas. Most of the participants (90.9%) had been on ART treatment for at least 2 years. Before transitioning to DTG-based regimens, 784 children (65.7%) were on non-nucleoside reverse transcriptase inhibitor (NNRTI)-based ART, and 267 children (22.4%) were on protease inhibitor (PI)-based ART (see Table 1).

**TABLE 1:** Demographic characteristics of children living with HIV on dolutegravir-based regimens with high viral load results following routine testing in selected Elizabeth Glaser Pediatric AIDS Foundation-supported facilities, January 2022 to March 2023 (*N* = 1475).

Characteristics	*n*	%
**Gender**		
Female	725	49.2
Male	750	50.8
**Age range (years)**		
< 5	175	11.9
5–9	416	28.2
10–14	884	59.9
**Duration on ART (years)**		
< 2	134	9.1
≥ 2	1341	90.9
**Facility location**		
Rural	1008	68.3
Urban	467	31.7
**Initial ART regimen**		
NNRTI-based	784	65.7
PI-based	267	22.4
Dolutegravir-based	142	11.9
Missing	282	-
**Enrolled in EAC**		
Yes	1448	98.2
No	27	1.8
**Number of EAC sessions completed**		
1	353	24.4
2	308	21.3
3	787	54.3
**Post-EAC VL test done**		
Yes	1091	75.4
No	357	24.6
**Post-EAC VL results**		
Suppressed	782	71.7
Not suppressed	309	28.3
**HIV DR test application sent to ARV committee**		
Yes	19	6.1
No	290	93.9
**HIV DR test approved**		
Yes	16	84.2
No	3	15.8
**HIV DR test performed**		
Yes	10	62.5
No	6	37.5
**Dolutegravir DR mutation found**		
Dolutegravir resistance	1	10.0
No dolutegravir resistance	9	90.0

Note: Age (years): Median = 11; IQR = 7–13.

IQR, interquartile range; ART, antiretroviral therapy; ARV, antiretroviral; DR, drug resistance; EAC, enhanced adherence counselling; PI, protease inhibitor; NNRTI, non-nucleoside reverse transcriptase inhibitor; VL, viral load.

A total of 1448 participants (98.2%) were enrolled in EAC. Among these, 787 (54.3%) completed three sessions, 308 (21.3%) completed two sessions, and 353 (24.4%) completed one session. Approximately three-quarters of participants who were enrolled in EAC (1091, 74%) underwent a follow-up HIV-1 viral load test, with 782 (71.7%) achieving VS (VS).

On multivariable analysis, patients from urban areas were less likely to achieve post-EAC compared to those from rural areas, with an adjusted odds ratio (aOR) of 0.58 (95% confidence interval [CI]: 0.42–0.80). No significant differences were observed in the likelihood of achieving VS based on age, gender, initial ART regimen, or the number of EAC sessions completed (Table 2).

**TABLE 2:** Factors associated with VS among paediatric antiretroviral therapy clients on dolutegravir-based regimens with high viral load results following enhanced adherence counselling in selected Elizabeth Glaser Pediatric AIDS Foundation-supported facilities, January 2022 to March 2023.

Characteristic	Viral suppression (Yes)		Univariable			Adjusted		
	*n*	%	OR	95% CI	*P*	OR	95% CI	*P*
**Gender**								
Female	395	72.7	Ref	-	-	1	-	-
Male	387	70.6	0.90	0.69–1.15	0.287	0.85	0.63–1.15	0.287
**Age range (years)**								
< 5	78	66.1	0.75	0.50–1.14	0.18	0.79	0.48–1.31	0.362
5–9	224	72.7	1.03	0.76–1.39	0.859	1.06	0.75–1.51	0.748
10–14	480	72.2	Ref	-	-	1	-	-
**Duration on ART (years)**								
< 2	58	65.2	0.72	0.45–1.14	0.157	1.02	0.54–1.95	0.943
≥ 2	724	72.3	Ref	-	-	1	-	-
**Facility location**								
Rural	542	75.7	Ref	-	-	1	-	-
Urban	240	64.0	0.57	0.44–0.75	<0.001	0.58	0.42–0.80	0.001
**Initial ART regimen**								
NNRTI-based	420	73.0	Ref	-	-	1	-	-
PI-based	133	71.5	0.93	0.64–1.34	0.682	1.08	0.72–1.61	0.711
Dolutegravir-based	67	66.3	0.73	0.46–1.14	0.167	0.75	0.47–1.20	0.236
**Number of EAC sessions completed**								
1	131	68.2	Ref	-	-	1	-	-
2	176	74.6	1.37	0.90–2.08	0.148	1.20	0.74–1.93	0.464
3	475	71.6	1.18	0.83–1.67	0.359	1.18	0.79–1.74	0.421

OR, odds ratio; CI, confidence interval; ART, antiretroviral therapy; EAC, enhanced adherence counselling; PI, protease inhibitor; NNRTI, non-nucleoside reverse transcriptase inhibitor; Ref, reference.

Among children who did not achieve VS, despite successful EAC, 19 (6.1%) had applications for HIV DR testing sent to the national ARV committee; 16 applications (84.1%) were approved. Of those approved, 10 (62.5%) had HIV DR testing, and one (10.0%) was found to have resistance to DTG.

## Discussion

Our study provides data on the management of CLHIV on ART with suspected treatment failure, with a particular focus on EAC in the era of DTG-based ART, in a programme setting in Malawi. Notably, a significant proportion (28.7%) of paediatric ART clients on DTG-based regimens remain virally non-suppressed following EAC. CLHIV residing in urban areas were less likely to achieve VS post-EAC compared to those in rural areas.

EAC is a crucial intervention for patients with high viral loads, particularly in low-resource settings where HIV drug resistance testing is limited. Nearly all children in our cohort were enrolled in EAC, which is essential for managing those with suspected treatment failure. The high enrolment rate is consistent with studies from Uganda, Ethiopia, and Kenya.^27,28,29^ However, nearly a quarter of the patients enrolled did not have documented viral load results following EAC. We hypothesise that some children were lost to follow-up before going through the entire management cascade. Additionally, challenges with viral load coverage could have contributed to the drop-offs along the cascade. Addressing these issues is crucial for the continued success of the programme.

Most participants (71.7%) with post-EAC viral load results achieved VS, aligning with findings from other studies and the WHO recommendations.^18,19^ Studies have reported better VS rates following EAC in patients on DTG-based ART than those on PI-based or NNRTI-based regimens.^27,30^ The high re-suppression rates following EAC observed in our study further uphold the importance of good ART adherence to achieve VS among CLHIV, particularly those on DTG-based regimens. Similarly, Mhlanga et al. in Zimbabwe found that CLHIV receiving ART had a higher likelihood of viral non-suppression following EAC when compared to adults.^31^ Addressing challenges to ART adherence in children and adolescents – such as lack of awareness of HIV status, reliance on caregivers, and school-related barriers – is essential for improving VS in this group.^31,32,33,34,35^

Patients residing in urban areas were less likely to achieve VS following EAC than those residing in rural areas. Ng’ambi et al. also reported a lower likelihood of VS among ART patients in urban areas compared to their rural counterparts in Malawi.^36^ The higher burden of HIV in urban areas is characterised by a higher prevalence of the virus, a lower proportion of individuals aware of their HIV status and initiated onto treatment, and lower suppression rates compared to rural areas.^22^ Further research is needed to explore the barriers to HIV care among urban residents in Malawi.

Our study found no differences in the odds of post-EAC VS between children who completed one or two counselling sessions and those who completed three sessions. This aligns with findings from a retrospective study of HIV-positive patients with initial high viral load in Zimbabwe.^37^ It is important to note that the number of EAC sessions required for each patient was determined by providers based on their assessment of the quality and effectiveness of previous sessions. While Malawi’s Ministry of Health guidelines recommend a minimum of one quality EAC session for each patient, our finding supports the guidance’s emphasis on identifying and addressing all barriers to adherence with the patient rather than the frequency of the sessions.^25^

Only a very small proportion (6.1%) of the children who did not achieve VS post-EAC had their requests sent for HIV DR testing. Despite the limited resources for DR testing in Malawi, our study demonstrated that these resources were being underutilised. Interventions to improve awareness and implementation of the guidance on DR testing are necessary to improve its coverage. Among the 10 eligible paediatric ART patients who underwent DR testing, one had resistance to DTG. Further research is needed to adequately assess the burden and outcomes of HIV DR among children in Malawi in the era of DTG-based regimens. Addressing these key challenges and building on our findings would enhance the management of paediatric patients with suspected treatment failure and improve the overall ART outcomes in this group.

We acknowledge some limitations of our study. Use of programme data presents a higher chance of documentation error and missing data than in a controlled setting. Also, our study could not assess other associated factors like duration to complete EAC and caregiver characteristics, as such data are not routinely collected. Nonetheless, we believe our approach best evaluates paediatric HIV high viral load management in public facilities in Malawi.

## Conclusion

Our results show that nearly three-quarters of children with high viral load on dolutegravir-based regimens achieved VS following EAC. EAC remains a useful tool in the management of paediatric patients with treatment failure. Further research on other contributors to virologic failure among children in Malawi is required.
